# Measurement properties of the Spinal Appearance Questionnaire in adolescents with idiopathic scoliosis: a systematic review

**DOI:** 10.1186/s12891-023-06708-z

**Published:** 2023-07-18

**Authors:** Lorenna Costa Malaquias, Maria Clara Monteiro da Silva, Dhara Yasmin Andrade Menezes, Maurício Oliveira Magalhães

**Affiliations:** 1 Post Graduation Program in Human Movement Sciences, Federal University of Pará (UFPA), Belém, Pará Brazil; 2 Faculty of Physical and Occupational Therapy, Institute of Health Sciences, Federal University of Pará (UFPA), Belém, Pará Brazil; 3 Post Graduation Program in Human Movement Sciences, Federal University of Pará (UFPA), Belém, Pará Brazil

**Keywords:** Scoliosis, Physical appearance, Questionnaires and surveys, Patient reported outcome measures, Systematic review

## Abstract

**Background:**

Scoliosis is defined as a three-dimensional deformity of the spine characterized by lateral tilt and axial rotation of the vertebrae. Its magnitude in the frontal plane is identified by a Cobb angle greater than 10^o^. The aim of the study was to systematically examine the clinimetric properties of the Spinal Appearance Questionnaire (SAQ) in its cross-cultural adaptations in different languages.

**Methods:**

Medline (PubMed), CINAHL, EMBASE, Science Direct, PsycINFO and WorldWideScience.org databases were used for screening studies until July 16, 2022. In this study, records on the development, evaluation and translation of the SAQ instrument in adolescents with idiopathic scoliosis were included. In addition, two independent reviewers defined whether the studies were eligible and analyzed their psychometric properties of internal consistency, reliability, content validity, cross-cultural validity, construct validity and structural validity, according to the COnsensus-based Standards for the selection of health Measurement INstruments (COSMIN). The modified GRADE was applied for evidence synthesis.

**Results:**

A total of 95 articles were selected by title and abstract. After removing duplicates and reading and searching the references, a total of 13 studies were included in this review. The original version of the SAQ was described in English, and the instrument was translated into Polish, Canadian French, Simplified Chinese, Spanish (Europe), Danish, Traditional Chinese, Portuguese (Brazil), Korean, German, Turkish and Persian. The evidence was moderate for construct validity, low for internal consistency, and very low for reliability and cross-cultural validity; the content and structural validity properties did not present minimum data for classification.

**Conclusion:**

The quality of the evidence regarding the clinimetric properties of the SAQ instrument in adolescents with idiopathic scoliosis was low due to the absence of clinimetric properties or dubious methodological quality. However, for clinical practice and research, we recommend the use of the instrument to assess the self-perception of the spine in adolescents. For future translations and adaptations, we recommend the use of the COSMIN guidelines.

**Supplementary Information:**

The online version contains supplementary material available at 10.1186/s12891-023-06708-z.

## Background

Adolescent idiopathic scoliosis (AIS) is the most common form of scoliosis, affecting approximately 0.47 and 5.2% of individuals with scoliosis worldwide, and is associated with high health costs [1–3]. Scoliosis is defined as a three-dimensional deformity of the spine characterized by lateral tilt and axial rotation of the vertebrae. Its magnitude in the frontal plane is identified by a Cobb angle greater than 10°. The literature has shown a greater predominance in females, and its progression is more tangible at the growth peak that occurs at puberty, between 11 and 14 years of age [4, 5].

The progression of the scoliosis curve during adolescence can be marked by changes in respiratory and functional status, pain intensity and aesthetic appearance. Concern about appearance is associated with worse health-related quality of life, and this one of the main reasons for referral to health care professionals [6–8]. In addition, scoliosis can strongly affect self-image, mental health and activities of daily living [6, 9]. In recent years, understanding of health and disability has increased, with greater emphasis placed on evaluation and treatment measures related to quality of life in this population [10, 11].

The therapeutic process of AIS is characterized by aligning the expectations and goals of the patient regarding treatment. One way to evaluate treatment is via patient-reported outcome measures (PROMs) [12]. Based on the self-report of patients and measured by instruments, there are data on, for example, adolescents’ perception of their appearance and expectations about their image of adolescents with idiopathic scoliosis. In this context, the Spinal Appearance Questionnaire (SAQ) is a validated instrument that measures the perception of spine appearance and deformity and AIS patient expectations about self-image [13–15].

The original version of the SAQ in English was developed by Sanders et al. (2007) with seven design items for the indication of spinal deformities and the progression of severity. Subsequently, it was improved by Carreon et al. (2011), who demonstrated the SAQ as a tool with greater sensitivity for self-image when compared to the *Scoliosis Research Society-22* (SRS-22) [16, 17] Thus, the clinical practice guidelines recommend that health professionals use instruments adapted and validated cross-culturally with adequate methodological quality. However, there are no studies that have grouped and evaluated the properties of the SAQ in a systematic way and analyzed its degrees of recommendation based on the COnsensus-based Standards for the selection of health Measurement INstruments (COSMIN) [18–20]. Thus, the objective of this study was to systematically examine the clinimetric properties of the SAQ instrument in its cross-cultural adaptations in different languages.

## Methods

This systematic review was registered in the *International Prospective Register of Systematic Review* (PROSPERO), CRD42021250114. The search, writing and systematic review strategies were developed according to the recommendations of the *Preferred Reporting Items for Systematic Reviews and Meta-Analyses* (PRISMA) [21, 22].

Studies that used or examined the SAQ and reported data regarding the clinimetric properties of the questionnaire in different languages were eligible. There were no time restrictions, and the inclusion of studies was primarily based on the main outcomes of translation, adaptation and validation of the instrument in clinical or academic contexts. Incomplete studies, those that were limited to analyses of adults or those that used the SAQ with spinal deformities other than AIS were excluded.

The databases Medline (PubMed), CINAHL, EMBASE, Science Direct, PsycINFO and WorldWideScience.org were used to screen studies until July 16, 2022. The search strategy consisted of three groups of search terms combined with the Boolean operator ‘AND’, represented by the following components: [1] Scoliosis, [2] Adolescent and [3] Spinal Appearance Questionnaire measurement properties (e.g., reliability, validity, responsiveness). The complete search strategies adapted to each database are described in Appendix A. The search descriptors were limited to English and human studies. The searches and selection of articles were performed by two independent reviewers in the databases (MCMS and DYAM). In case of discrepancies, a third reviewer mediated (LCM).

Initially, there was a screening to assess the suitability of the articles per the inclusion criteria based on the titles and abstracts, followed by a complete reading of the selected articles. Eligible articles were assessed for methodological quality. The measurement properties were divided into three domains: reliability (including internal consistency, reliability and measurement error), validity (including content validity, construct validity and criterion validity), and responsiveness. It is noteworthy that the split construct validity can present itself with the properties of structural validity, hypothesis testing and cross-cultural validity. Thus, the “*Consensus-based Standards for the selection of health Measurement INstruments* (COSMIN)” were used to assess methodological quality based on the COSMIN protocol for systematic reviews of measurement properties [20]. This stage with the included studies was performed by two independent authors, and consensus was reached in meetings with the third author when necessary.

The methodological quality rating was first determined by collecting data on PROM characteristics. The population included the results of measurement properties and information on scores from that PROM in each study. In sequence, quality classification was determined to be very good, adequate, doubtful, or inadequate in each study by measurement property (cross-cultural adaptation, internal consistency, reliability, error measure, responsiveness, content validity, structural validity, criterion validity and validity of construct) were compared to the results of classification for good measurement properties and classified as sufficient (+), insufficient (-) or indeterminate (?). In addition, the analyses allow a grouped result of the measurement properties with general classifications of sufficient (+), insufficient (-), inconsistent (±) or indeterminate (?) and the classification of the quality of evidence of these properties, as proposed by the COSMIN manual, according to the modified *Grading of Recommendations Assessment, Development and Evaluation* (GRADE) scale, composed of stages: high, moderate, low, and very low [20].

The comparison instrument used was the SRS-22 considered the most robust and specific to individuals with adolescent idiopathic scoliosis, having a domain of spine self-image and used with the comparator instrument in all studies included in this work. Thus the hypothesis of this study is based on a comparison of the SAQ and the self-image dimension of the *Scoliosis Research Society-22* (SRS-22) and the premise that this would show a correlation between the instruments with similar constructs of low or fair aspects (0.25–0.50) [20].

## Results

**Fig. 1:**
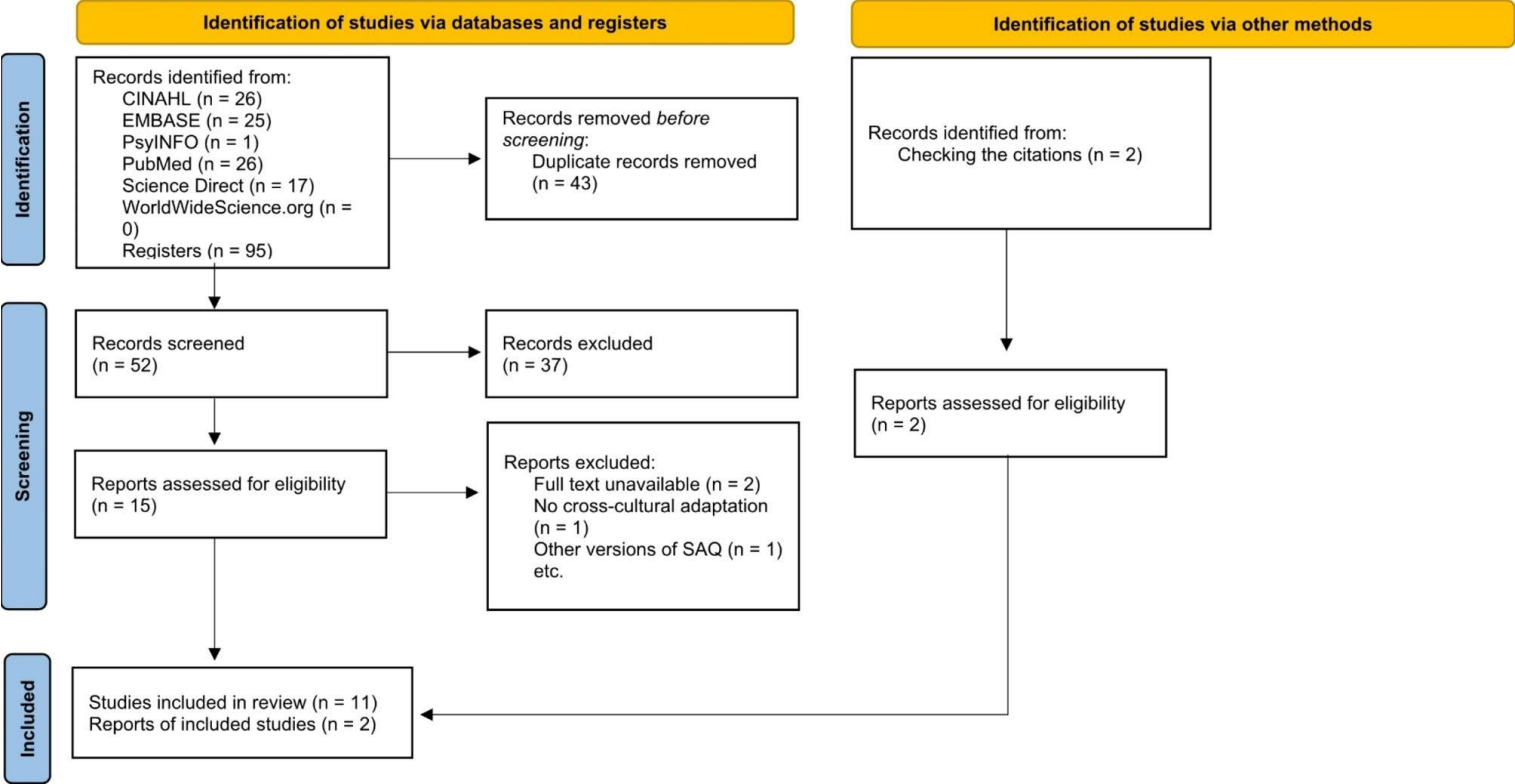
Flowchart with search steps

The search strategy resulted in 95 articles selected by title and abstract. After removal of duplicates, 52 studies were analyzed for inclusion, and 11 met the eligibility criteria. In addition, the follow-up of references resulted in two (n = 2) articles being included (Fig. 1).

The reasons for exclusion were as follows: full article not published or available (n = 2), articles that did not perform evaluation of cross-cultural adaptation or psychometric properties (n = 1) and another version of the SAQ was used for kyphosis (n = 1). Thus, the review was conducted with 13 articles that addressed the psychometric properties of the SAQ in 11 languages (Fig. 1).

The study population comprised 3,420 adolescents with AIS, the majority of whom were female (Table 1). The groups were organized by degrees of severity, Cobb angle and type of treatment. The description of the follow-up period of the participants occurred in five articles, with a mean of 14.6 ± 7.1 months [23–28].

**Table 1:** Sample characterization

Characterization of the disease							Instrument management			
Ref.	n	Age (years)	Female (%)	Degree of severity by Cobb angle (N)	Type of treatment (N)	Duration of treatment (months)	Scenario	Country	Language	Response rate
Sanders et al. (16)	G1: 127 G2: 235 G3: 83	-	-	G1: ≥10° (5) 11° − 20° (20) 21° − 30° (39) > 30° (63) G2 e G3: *	G1: No scoliosis (25) Observed (37) Orthosis (37) Recommended surgery (9) Postsurgical (19) G2 and G3: presurgical 1 year after surgery	-	-	United States	English	-
Carreon et al. (17)	1.802	14.8 ± 2.1	83	55, 8°±13,7° (0°-123°)	Observed (*) Braces (*) Presurgical (*)	-	-	United States	English	-
Misterka; Glowack; Harasymczuk (24)	40	15 ± 1,5	100	Preoperative: 55,3 (9,7) Postoperative: 29,1 (10,1)	Surgical	24	Department of Pediatric Orthopedics, Poznan University of Medical Sciences	Poland	Polish	-
Roy-beaudry et al. (29)	182	15 ± 2,5	88,5	≥ 10° (18) 11° − 20° (54) 21° − 30° (50) > 30° (60)	Observation (124) Orthosis (18) Presurgical (8) Postsurgical (32)	-	Scoliosis Clinics at Sainte Justine University Hospital Center	Canada	French Canadian	-
Wei et al. (25)	215	14,58 ± 1,58	85,6	10° − 25° (81) 25° − 40° (56) 40° − 55° (34) 55° − 70° (19) 70° − 85° (18) 85° − 100° (7)	Excercise (77) Orthosis (66) Surgery (72)	22	Orthopedics Outpatient Clinic of the Changai Hospital of the Second Military Medical University	China	Simple Chinese	-
Matamalas et al. (30)	80	< 18 (13, 9 ± 1,9) ≥ 18 (27, 3 ± 7,4)	< 18 85,7 ≥ 18 84,2	< 45° (ǂ35.2°) (40) ≥ 45° (ǂ 56.6°) (40)	-	-	Vall d’Hebron Hospital	Spain	Spanish (Europe)	-
Simony et al. (31)	51	16 ± 3	-	-	Boston orthosis (*) Surgical (*)	-	Odense University Hospital	Denmark	Danish	-
Guo et al. (26)	112	17,3 ± 3,1	100	10°–19° 20°–29° 30°–39° 40°–49° 50°	Observation (29) Orthosis (16) Pós-Orthosis (45) Pós-operatório (22)	09	Joint Scoliosis Research Center of the Chinese University of Hong Kong and Nanjing University	China	Traditional Chinese	-
Rosendo et al. (32)	20	14,8 ± 2,5	90	-	Surgery (4) Observation (16)	-	Hospital Getúlio Vargas	Brazil	Portuguese (Brazil)	-
Lee et al. (33)	112	12 ± 1,4	88,4	29,1° ± 13,2°	Observation (32) Orthosis (53) Surgery (27)	-	-	South Korea	Korean	-
Thielsch et al. (27)	255	30,0 ± 16,7	85,1	43,5° ± 20,9°	Physiotherapy (*) Braces (*) Surgery (*)	12	Department of Orthopedics, University Hospital of Münster	Germany	German	-
Yapar et al. (28)	75	15,5 (10–18)	74,7	37,6° (13° − 76°)	Braces (*) Surgery (*)	6	Department of Orthopedics and Traumatology, Ankara	Turkey	Turkish	-
Babaee et al. (9)	106	13,6 ± 1.8	73,59	39,35°±12.1	Observation (19) Orthosis (72) Postbracing (awaiting surgery) (15)	-	Department of Orthotics and Prosthetics, School of Rehabilitation Sciences, University of Medicine of Iran	Iran	Persian	-

G1 = Group 1; G2 = Group 2; G3 = Group 3

*Number of participants not reported

ǂ Mean

- No report of the variable

### Methodological quality

The properties evaluated in this review were internal consistency, reliability, content validity, cross-cultural validity, construct validity and structural validity (Table 2). There is a description of the number of participants, aspects of methodological quality and a classification for good measurement properties (Table 3).

**Table 2:** Methodological quality in each study by measurement property (COSMIN Checklist)

STUDY	LANGUAGE	INTERNAL CONSISTENCY	RELIABILITY	CONSTRUCTION VALIDITY	MEASUREMENT ERROR	CONTENT VALIDITY	STRUCTURAL VALIDITY	HYPOTHESIS TEST	CROSS-CULTURAL VALIDITY	VALIDITY OF CRITERIA	RESPONSIBILITY
Sanders et al. (16)	English	Doubtful	Inadequate	Doubtful		Inadequate	Inadequate				
Carreon et al. (17)	English	Very good	Inadequate	Inadequate			Inadequate				
Misterka; Glowack; Harasymczuk (24)	Polish	Very good	Inadequate				Inadequate		Inadequate		
Roy-beaudry et al. (29)	French Canadian	Doubtful	Inadequate	Adequate			Inadequate		Inadequate		
Wei et al. (25)	Simplified Chinese	Doubtful	Inadequate	Adequate			Inadequate		Inadequate		
Matamalas et al. (30)	European Spanish	Very good	Doubtful	Doubtful			Inadequate		Inadequate		
Simony et al. (31)	Danish	Very good	Inadequate	Doubtful			Inadequate		Inadequate		
Guo et al. (26)	Traditional Chinese	Very good	Doubtful	Very good			Doubtful		Doubtful		
Rosendo et al. (32)	Portuguese (Brazil)	Inadequate	Inadequate				Inadequate		Inadequate		
Lee et al. (33)	Korean	Inadequate	Adequate	Adequate			Inadequate		Inadequate		
Thielsch et al. (27)	German	Very good	Inadequate	Doubtful			Inadequate		Doubtful		
Yapar et al. (28)	Turkish	Very good	Inadequate	Doubtful			Inadequate		Inadequate		
Babaee et al. (9)	Persian	Very good	Inadequate	Very good			Inadequate		Doubtful		

**Table 3:** Grouped results of the SAQ measurement properties with overall classification (COSMIN Checklist)

**STUDY**	Structural validity			Internal consistency			Reliability			Cross-cultural validity			Construct validity		
	**n**	Method	**Classification**	n	Method	Classification	n	Method	Classification	n	Method	Classification	n	Method	Classification
Sanders et al. (16)	127	Inadequate	?	127	Doubtful	α Cronbach = > 0.7 (+)	93	Doubtful	*r* = 0.57–0.99 (?)	*	*	*	235	Doubtful	*
Carreon et al. (17)	1.802	Inadequate	-	1.802	Very good	α Cronbach = 0.88 (+)	1.802	Inadequate	α Cronbach = 0.89 (?)	*	*	*	1.082	Inadequate	*r*_*s*_ = − 0.43 *P* < 0.0001 (+)
Misterka; Glowack; Harasymczuk (24)	40	Inadequate	-	40	Very good	α Cronbach = 0.91 (+)	40	Inadequate	*r* = 0.98 (?)	40	Inadequate	?	*	*	*
Roy-beaudry et al. (29)	182	Inadequate	-	182	Doubtful	*r* = 0.34–0.76 P = 0.01 (?)	182	Inadequate	*r* = 0.34–0.76 P = 0.01 (?)	182	Inadequate	?	182	Adequate	*r*_*s*_ < 0.36 *P* < 0.01 (+)
Wei et al. (25)	215	Inadequate	-	215	Doubtful	ICC = 0.52–0.80 (P < 0.0001) (?)	215	Inadequate	ICC = 0.93 (+)	215	Inadequate	?	215	Adequate	*r*_*s*_ = − 0.40 *P* < 0.0001 (+)
Matamalas et al. (30)	80	Inadequate	-	80	Very good	α Cronbach = 0.88 (+)	80	Doubtful	α Cronbach = 0.88 (?)	80	Inadequate	?	80	Doubtful	ρ = − 0.67 (+)
Simony et al. (31)	51	Inadequate	-	51	Very good	α Cronbach = 0.89 (+)	51	Inadequate	ICC = 0.89 (+)	51	Inadequate	?	51	Doubtful	*r*_*s*_ = − 0.60 (+)
Guo et al. (26)	112	Doubtful	-	112	Very good	α Cronbach = 0.78–0.94** (+)	112	Doubtful	ICC = 0.79–0.86** (?)	101	Doubtful	?	101	Very good	ρ = 0.41 *P* < 0.0001 (+)
Rosendo et al. (32)	20	Inadequate	-	20	Inadequate	α Cronbach = 0.79 (+)	20	Inadequate	α Cronbach = 0.79 (?)	20	Inadequate	?	*	*	*
Lee et al. (33)	112	Inadequate	-	112	Inadequate	α Cronbach = 0.88 (+)	112	Adequate	ICC = 0.92 (+)	112	Inadequate	?	112	Adequate	ρ = − 0.45 *P* < 0.001 (+)
Thielsch et al. (27)	255	Inadequate	-	255	Very good	α Cronbach = 0.91 (+)	113	Inadequate	α Cronbach = 0.80 (?)	255	Doubtful	?	255	Doubtful	*r*_*s*_ = − 0.60 *P* < 0.01 (+)
Yapar et al. (28)	75	Inadequate	-	75	Very good	α Cronbach = 0.91 (+)	75	Inadequate	ICC = 0.98 (+)	75	Inadequate	-	75	Doubtful	*r*_*s*_ = − 0.60 *P* < 0.001 (+)
Babaee et al. (9)	106	Inadequate	-	106	Very good	α Cronbach = 0.77 (+)	38	Inadequate	ICC = 0.98 (+)	106	Doubtful	-	106	Very good	*r*_*s*_ = 0.40 *P* < 0.01 (+)
Grouped result (general classification)	3.177		-	3.177		αCronbach = > 0.7–0.94 (+)	2.933		ICC = 0.89–0.98 (+)	1.237		-	2.494		*r*_*s*_ = 0.36 ρ = 0.41–0.67 +

Abbreviations: Qual Method: Methodological quality, Results: result with classification,

“+” = sufficient, “–” = insufficient, “?” = undetermined

Grouped result: “+” = sufficient, “–” = insufficient, “±” inconsistent, “?” = undetermined

ICC = Intraclass correlation coefficient

ρ = Pearson’s correlation coefficient

***r***_***s***_ = Spearman’s rank correlation coefficient

*The analysis was not performed

**Data presented for variation in each domain. The study did not describe the total value of the scale

The original version was developed in English by Sanders et al. (2007) [16] and later improved by Carreon et al. (2011) [17]. All other versions of the instrument had only one publication in each language. The SAQ has been translated into Polish [24], French Canadian [29], Simplified Chinese [25], Spanish (Europe) [30], Danish [31], Traditional Chinese [26], Portuguese (Brazil) [32], Korean [33], German [27], Turkish [28] and Persian [9].

Internal consistency was tested with a population of 3,177 participants, and its methodological quality was determined in 8 articles as “very good”, 3 “doubtful” and 2 “inadequate”; the pooled classification result was “sufficient” with Cronbach’s alpha values of 0.70–0.94 in 11 studies [16, 17, 24, 26–28, 30–33].

Reliability was tested with a population of 2,933 participants, and its methodological quality was determined to be “adequate” in 1 article, “doubtful” in 3 and “inadequate” in 9. The pooled classification result was “sufficient”, with intraclass correlation coefficients (ICCs) of 0.89–0.98 in 5 studies [9, 25, 28, 31, 33].

Content validity was tested with a population of 127 participants only in the original article [16], and its methodological quality was “inadequate” with insufficient data for continuing analysis.

The cross-cultural validity was tested with a population of 1,237 participants, and its methodological quality was presented as “doubtful” in 3 articles [9, 26, 27] and “inadequate” in 8 articles [24, 25, 28–33]. The grouped classification result was “insufficient”, without correct statistical tests to test the populations.

Structural validity was tested in a population of 3,177 participants and found to be of “doubtful” methodological quality in 1 article [16] and “inadequate” in 12 articles. The grouped result was “indeterminate”, as the authors of the study diverged from the group and demonstrated one of the statistical tests necessary for the correct correlation of the data.

The construct validity was evaluated in 11 articles and a total population of 2,494 people with a methodological quality of “inadequate” in 1 article [17], “doubtful” in 5 articles [16, 27, 28, 30, 31], “adequate” in 3 articles [25, 29, 33] and “very good” in 2 articles [9, 26].

## Discussion

The present study aimed to systematically review the clinimetric properties of the Spinal Appearance Questionnaire (SAQ) in adolescents with idiopathic scoliosis and analyze its cross-cultural adaptations according to the COSMIN guidelines [34]. The main results suggest that the 13 articles included in this review have methodological inconsistencies regarding psychometric properties and, especially, among statistical tests.

The evidence suggests that the SAQ, in its version for patients, presented a modified quality of evidence (GRADE) of moderate for construct validity, low for internal consistency, and very low for reliability and cross-cultural validity; the content and structural validity properties did not present sufficient data for classification.

The unidimensional structure of the questionnaire was confirmed in most articles with populations exclusively of adolescents [9, 15–17, 24–26, 28, 29, 31–33]. According to the modified GRADE analysis, only two studies reported their results associating groups of adolescents and adults with scoliosis, a fact that added indirect risk of bias due to the partial use of other populations [27, 30].

For a better understanding of these analyses carried out, the measurement properties were arranged in topics from the domains mentioned in reliability (including internal consistency, reliability and measurement error), validity (including content validity, construct validity and criterion validity), and responsiveness.

### Reliability

Internal consistency is defined as the extent to which the items of a scale or subscale of the questionnaire are correlated, measuring the same construct. As a measurement property, it is an important requirement for one-dimensional instruments, which aim to measure a single construct using several items, as in the case of the SAQ. Its evaluation is given by Cronbach’s alpha, a coefficient that reflects the degree of covariance between the items of a scale, with compliance parameters between 0.70 and 0.95 [19, 35].

During the evaluations of this study, 11 articles [9, 16, 17, 24, 26–28, 30–33] with grouped Cronbach’s alpha values between 0.70 and 0.94 and a classification of “sufficient” according to COSMIN were obtained. The two other articles did not perform analyses with adequate statistical tests to measure internal consistency [25, 29]. However, the modified GRADE was considered low due to the risk of bias being estimated as “serious” due to the indirect risk of population bias and the number of studies with inadequate or dubious methodological quality [34].

Reliability is part of an expression of the stability of the reproducibility of the instruments with different people (test-retest) that allow similar, coherent and precise responses. Among the reliability coefficients, the ICC is most appropriate for the evaluation of continuous measures. For this property, Pearson’s correlation coefficient is inadequate because systematic differences are not taken into account. For ordinal measures, the weighted Cohen Kappa coefficient should be used. In the case of ICC or weighted Kappa, a minimum standard of 0.70 is recommended for good reliability and in a sample of at least 50 people [35, 36].

The reliability property was evaluated in four studies [9, 25, 28, 33], which demonstrated correct approaches to coefficients. The present study observed pooled results of ICC between 0.92 and 0.98 and “sufficient” classification according to COSMIN [34]. However, of the 13 articles analyzed in this study, nine presented incompatibilities, such as in the statistical tests of the measures evaluated, failure to consider systematic differences in their populations and the minimum sample size. In addition, the modified GRADE quality classification was considered very low due to a “very serious” risk of bias because there was only one study with “adequate” quality [34].

### Validity

Cross-cultural validity refers to the degree to which the performance of the items in a translated or culturally adapted instrument reflects the original version of the instrument. To assess cross-cultural validity, property measurement data from at least two different groups are required for comparison, with differences such as gender or language [34]. However, none of the studies included in this study observed the achievement of this fundamental point of the COSMIN. This fact made it impossible to estimate the quality of evidence.

The process of translation and adaptation of instruments refers to the resolution of differences in customs, language and perception of health between different countries and cultures, allowing comparisons between different populations and exchange of information across linguistic and cultural barriers [37, 38]. In this regard, all studies followed the international guidelines recommended by Beaton et al. (2000) [37] presenting translated versions of the SAQ in Polish, Canadian French, Simplified Chinese, European Spanish, Danish, Traditional Chinese, Brazilian Portuguese, Korean, German, Turkish and Persian [16, 17, 24, 26–28, 30–33, 39].

Structural validity refers to the degree to which the patient-reported outcome (PROM) scores are an adequate reflection of the dimensionality of the construct to be measured [40]. However, only one study performed the evaluation of structural validity [16], and the authors tested the property by means of standardized fit statistics with z scores and with the absence of other standard tests expected by the COSMIN, such as item response theory. Due to the inconsistency of the results presented in this property, the modified GRADE classification was not performed [34].

Criterion validity refers to the degree to which the scores of a PROM are an adequate reflection of the gold standard [34]. The review team of this article decided not to address the suggested tables for the evaluation of the COSMIN criterion validity and responsiveness, as these two properties are based on comparisons with a gold standard for health status questionnaires in the target population. This fact is not possible with the SAQ due to its illustrative design, which is a characteristic of the instrument. However, all evidence of the validity of the articles will be included in the analyses of construct validity [41, 42].

Construct validity reflects the ability of an instrument to measure the theoretical dimensions of a construct. As abstract constructions do not manifest themselves directly as physical events, their inferences may derive from observable behaviors and patient self-report. For COSMIN, the construct validity is assessed through the hypothesis test, where the consistency of the scores of a PROM are estimated from the comparison of instruments. Thus, the more specific the hypotheses and the more hypotheses tested, the more evidence is collected for this measurement property [40].

The property of construct validity can be observed in the articles through the subtopic of convergent validity. In this systematic review, the hypothesis was established that correlations should vary from weak to moderate (***r =*** 0.25 to 0.50) [23]. Eleven articles performed the association and description of the variables following the appropriate correlation tests, such as Spearman and Pearson, obtaining a clustered result of “sufficient” for the measurement property of the SAQ. In addition, the modified GRADE quality classification was considered “moderate” due to the indirect risk of population bias [34]. The articles analyzed during the review were compared using the SRS-22 instrument’s self-image domain, which is aimed at teenagers and has had its psychometric structures extensively tested.

Content validity is the degree to which the content of an instrument is an adequate reflection of the construct to be measured and is of interest to the target population. This property should systematically involve patients and professionals in the field to achieve aspects of relevance, scope and understandability of the items. The definition of the target population and the context in which the instrument is used are important aspects for the evaluation of content validity, and it is recommended to perform this evaluation only in its original version [21].

Thus, during the evaluation of the original article of the instrument [16], the measured construct and evaluative context are well described; however, its target population is not clearly cited, involving only adolescents with AIS. Furthermore, the participants are not included in the process of constructing the questionnaire together with experts. Thus, the lack of comprehensiveness and comprehensibility tests as recommended by COSMIN resulted in the methodological quality being classified as “inadequate”. Regarding the other studies, this property was not evaluated; thus, the GRADE classification of this psychometric property was not performed.

### Strengths and limitations

The strengths of the study are that the analyses performed were in accordance with the most recent guidelines of the COSMIN manual for systematic reviews. Thus, the systematic review was based on a broad investigation of articles that addressed the SAQ in adolescents. In addition to preventing data loss, all articles analyzed the psychometric properties of the instrument, including its original version.

However, the limitations observed in this study were that grouped data of the articles present discrepancies that can lead to a false grouping of the data for the measured property. Therefore, this article does not present data on a meta-analysis of the data. Another point was the predilection for the use of English in database searches and obtaining articles transcribed in English.

## Conclusion

After extensive investigation of the clinimetric properties of the Spinal Appearance Questionnaire instrument in its cross-cultural adaptations, the quality of the evidence regarding the questionnaire in adolescents with idiopathic scoliosis was low due to the absence of clinimetric properties or dubious methodological quality. However, for clinical practice and research, we recommend the use of the instrument to assess the self-perception of the spine in adolescents. For future translations and adaptations, we recommend the use of the COSMIN guidelines.

## Electronic supplementary material

Below is the link to the electronic supplementary material.

Appendix A

## Data Availability

The datasets generated and/or analyzed during the current study are not publicly available due these data will still be used as auxiliary data in our other studies and we do not wish to publish them publicly but they are available from the corresponding author on reasonable request.
