# Supplementary material for: Measurement properties of the Spinal Appearance Questionnaire in adolescents with idiopathic scoliosis: a systematic review
Source: BMC Musculoskelet Disord. 2023 Jul 18;24:587. doi: 10.1186/s12891-023-06708-z (PMC10354899; doi:10.1186/s12891-023-06708-z)
Supplement: Supplementary file 1 — Appendix A [file 12891_2023_6708_MOESM1_ESM.docx]

**APPENDIX A – Complete search strategy in each database**

| Database | Search strategy |
| --- | --- |
| PubMed | “Scoliosis” OR “Idiopathic Scoliosis” OR “AIS” OR “Adolescent Idiopathic Scoliosis” AND “Adolescent” OR “Adolescents” OR “Adolescence” OR “Teens” OR “Teen” OR “Teenagers” OR “Teenager” OR “Youth” OR “Youths” OR (“Adolescents”, “Female”) OR (“Adolescent”, “Female”) OR (“Female Adolescent”) OR (“Female Adolescents”) OR (“Adolescents”, “Male”) OR (“Adolescent”, “Male”) OR (“Male Adolescent”) OR (“Male Adolescents”) AND “Spinal Appearance Questionnaire” OR “SAQ” AND “Reliability” OR “consistency” OR “validity” OR “responsiveness” OR “ calibration” OR “validation” OR “agreement” OR “minimal detectable change” OR “clinically important difference” OR “psychometric properties” OR “measurement properties” |
| CINAHL | “Scoliosis” OR “Idiopathic Scoliosis” OR “AIS” OR “Adolescent Idiopathic Scoliosis” AND “Adolescent” OR “Adolescents” OR “Adolescence” OR “Teens” OR “Teen” OR “Teenagers” OR “Teenager” OR “Youth” OR “Youths” OR (“Adolescents”, “Female”) OR (“Adolescent”, “Female”) OR (“Female Adolescent”) OR (“Female Adolescents”) OR (“Adolescents”, “Male”) OR (“Adolescent”, “Male”) OR (“Male Adolescent”) OR (“Male Adolescents”) AND “Spinal Appearance Questionnaire” OR “SAQ” AND “Reliability” OR “consistency” OR “validity” OR “responsiveness” OR “ calibration” OR “validation” OR “agreement” OR “minimal detectable change” OR “clinically important difference” OR “psychometric properties” OR “measurement properties” |
| BASELINE | 'Scoliosis' OR 'Idiopathic Scoliosis' OR 'AIS' OR 'Adolescent Idiopathic Scoliosis' AND 'Adolescent' OR 'Adolescents' OR 'Adolescence' OR 'Teens' OR 'Teen' OR 'Teenagers' OR 'Teenager' OR 'Youth' OR 'Youths' AND 'Spinal Appearance Questionnaire' OR 'SAQ' AND 'Reliability' OR 'consistency' OR 'validity' OR 'responsiveness' OR 'calibration' OR 'validation' OR 'agreement' OR 'minimal detectable change' OR ' clinically important difference' OR 'psychometric properties' OR 'measurement properties' |
| Science Direct | ("Scoliosis") AND ("Adolescent") AND ("Spinal Appearance Questionnaire") AND ("Reliability" OR "consistency" OR "responsiveness" OR "validation" OR "psychometric properties" OR "measurement properties") |
| PsychINFO | “Scoliosis” OR “Idiopathic Scoliosis” OR “AIS” OR “Adolescent Idiopathic Scoliosis” AND “Adolescent” OR “Adolescents” OR “Adolescence” OR “Teens” OR “Teen” OR “Teenagers” OR “Teenager” OR “Youth” OR “Youths” OR (“Adolescents”, “Female”) OR (“Adolescent”, “Female”) OR (“Female Adolescent”) OR (“Female Adolescents”) OR (“Adolescents”, “Male”) OR (“Adolescent”, “Male”) OR (“Male Adolescent”) OR (“Male Adolescents”) AND “Spinal Appearance Questionnaire” OR “SAQ” AND “Reliability” OR “consistency” OR “validity” OR “responsiveness” OR “ calibration” OR “validation” OR “agreement” OR “minimal detectable change” OR “clinically important difference” OR “psychometric properties” OR “measurement properties” |
| WorldWideScience.org | 'Scoliosis' AND 'Adolescent' AND 'Spinal Appearance Questionnaire' AND 'measurement properties' |

Source: Prepared by the author
